# Global Characterization of GH10 Family Xylanase Genes in *Rhizoctonia cerealis* and Functional Analysis of Xylanase RcXYN1 During Fungus Infection in Wheat

**DOI:** 10.3390/ijms21051812

**Published:** 2020-03-06

**Authors:** Lin Lu, Yongwei Liu, Zengyan Zhang

**Affiliations:** 1 Institute of Crop Sciences, National Key Facility for Crop Gene Resources and Genetic Improvement, Chinese Academy of Agricultural Sciences, Beijing 100081, China; lulin@caas.cn; 2 Institute of Genetics and Physiology, Hebei Academy of Agricultural and Forestry Sciences/Plant Genetic Engineering Center of Hebei Province, Shijiazhuang 050051, China; liuywmail@126.com

**Keywords:** cell death, pathogenicity, *Rhizoctonia cerealis*, wheat (*Triticum aestivum* L.), xylanases

## Abstract

Wheat (*Triticum aestivum L*.) is an important staple crop. *Rhizoctonia cerealis* is the causal agent of diseases that are devastating to cereal crops, including wheat. Xylanases play an important role in pathogenic infection, but little is known about xylanases in *R. cerealis*. Herein, we identified nine xylanase-encoding genes from the *R. cerealis* genome, named *RcXYN1–RcXYN9*, examined their expression patterns, and investigated the pathogenicity role of RcXYN1. RcXYN1–RcXYN9 proteins contain two conserved glutamate residues within the active motif in the glycoside hydrolase 10 (GH10) domain. Of them, RcXYN1–RcXYN4 are predicted to be secreted proteins. *RcXYN1–RcXYN9* displayed different expression patterns during the infection process of wheat, and *RcXYN1*, *RcXYN2*, *RcXYN5*, and *RcXYN9* were expressed highly across all the tested inoculation points. Functional dissection indicated that the RcXYN1 protein was able to induce necrosis/cell-death and H_2_O_2_ generation when infiltrated into wheat and *Nicotiana benthamiana* leaves. Furthermore, application of RcXYN1 protein followed by *R. cerealis* led to significantly higher levels of the disease in wheat leaves than application of the fungus alone. These results demonstrate that RcXYN1 acts as a pathogenicity factor during *R. cerealis* infection in wheat. This is the first investigation of xylanase genes in *R. cerealis*, providing novel insights into the pathogenesis mechanisms of *R. cerealis*.

## 1. Introduction

Wheat (*Triticum aestivum* L.) is one of the most important staple crops worldwide. Its highly efficient production is necessary for global food security [1]. However, wheat crops are subjected to numerous types of biotic and abiotic stresses, with fungal diseases representing one of the most serious threats to wheat production [2]. The necrotrophic fungus *Rhizoctonia cerealis* van der Hoeven, belonging to the binucleate *Rhizoctonia* subgroup AG-D I [3], is the causal agent of sharp eyespot, a disease mainly found on the stem base of wheat plants. Sharp eyespot can negatively impact both the quality and yield (∼10–40%) of wheat in many regions of Asia, Oceania, Europe, North America, and Africa [4,5]. Since the late 1990s, China has become the largest epidemic region, where more than 6.67 million hectares of wheat plants can be infected by *R. cerealis* annually [6,7,8]. Sharp eyespot can also occur on other cereal crops such as barley, oats, and rye [9,10]. In addition, the fungus can also infect other important economical crops and bio-energy plants, causing root rot disease in sugar beet, cotton, potato, and several legumes, and yellow patch in turf grasses [11,12]. To efficiently improve resistance of wheat and other plants to *R. cerealis*, it is necessary to explore the pathogenesis mechanism during the fungus–plant interactions.

The plant cell wall is a first obstacle, providing mechanical strength and rigidity to prevent microbial pathogen infection. The cell wall is composed of a complex network of various non-starch polysaccharides, lignin, and proteins [13]. In graminaceous monocotyledons and closely related monocot families, the components of the cell wall are rich in (glucurono-) arabinoxylans ((G)AXs, 20–40%) and mixed linkage glucans (10–30%), but contain only about 5% pectin [13]. To degrade cell wall polysaccharides such as pectins, hemicelluloses, and celluloses, microbial pathogens of plants have evolved an arsenal of cell wall-degrading enzymes (CWDEs), as reflected by the presence of abundant putative CWDE-encoding genes in their genomes [14,15,16]. These CWDEs are generally regarded as important virulence factors through the degradation of host macromolecules [17]. Xylan is the major component of hemicellulose, the second most abundant polysaccharide in nature [18]. Endo-*β*-1,4-xylanases (EC 3.2.1.8, xylanases), cleaving internal *β*-1,4-linkages between xylose units in AXs, are thus believed to be an important part of the offensive arsenal of microorganisms necessary to penetrate and colonize plant tissues [13]. Xylanases obtained from different microorganisms vary considerably in their primary sequences, specificity, and conformation, and have been classified into two major groups in the carbohydrate-active enzymes database (CAZy): glycoside hydrolase (GH) families 10 and 11 [19,20,21]. GH10 xylanases usually have a molecular mass of at least 30 kDa, whereas GH11 xylanases typically have a lower molecular mass (<30 kDa) [22]. Moreover, GH10 xylanases are generally less selective than GH11 xylanases and are able to attack decorated forms of the polysaccharide [22]. *Triticum aestivum* xylanase inhibitors (TAXI-type) are widely represented in cereal plants and specifically inhibit bacterial and fungal xylanases belonging to GH11 xylanases, but do not inhibit GH10 xylanases [13]. Therefore, it is speculated that members of the GH10 family may represent an important pathogenicity factor in the plant-pathogenic fungi infection of cereal crops.

Through attempts to elucidate the role of xylanases in microbial pathogenesis, variations in function were found [23]. Disruption of a Xyn11A of GH11 family from *Botrytis cinerea* had a profound effect on virulence, including delayed appearance of secondary lesions and a reduction inaverage lesion size by more than 70% [24]. In *Phytophthora parasitica,* ppxyn1 and ppxyn2, two xylanases from the GH10 family, play a crucial role in the pathogen infection process [25]. In *Septorianodorum*, xylan-degrading enzymes were highly induced when infecting *Graminaceous* monocotyledons, but the contribution of xylanases to pathogenicity is not clear [26,27]. Mutants of *Magnaportheoryzae* with a disruption in several xylanase genes can infect rice hosts as efficiently as the parent strains [28,29]. Similarly, target inactivation of two xylanase genes (*xyl3* and *xyl4*) in *Fusarium oxysporum* had no detectable effect on the fungal virulence in tomato plants [30]. A possible explanation for these results is the presence of a multigene family of xylanases in these fungi that may function redundantly in the infection process [29,31].

According to the lifestyles of plant pathogenic fungi, they are classified as biotrophs, hemi-biotrophs, and necrotrophs. Biotrophic pathogens have to obtain nutrients from living host cells and tissues, and often secrete limited amounts of CWDEs and effectors to suppress the host immune system [32]. In contrast, necrotrophic pathogens thrive on the dead host tissues that are killed before or during colonization. To induce cell death/necrosis, necrotrophic fungi often secrete phytotoxic secondary metabolites and peptides, and produce reactive oxygen species (ROS) [33]. Hemi-biotrophic pathogens display a biotrophic phase during early infection and display a necrotrophic phase only later; these pathogens produce toxins only at late stages in order to kill the host cells and complete their life cycle on dead tissues [33]. Genomic analysis of a necrotrophic*Rhizoctoniasolani*AG1-IA isolate identified three secreted effector proteins that were validated to elicit a cell death response when infiltrated into rice leaves [34].

To date, pathogenesis mechanisms in *R. cerealis* and the roles of xylanases in the fungal infection process on wheat have been largely unknown due to the absence of a whole genome sequence. Recently, we have completed the genome sequence, assembly, and annotation of *R. cerealis* isolate Rc207 (unpublished data). In this study, we globally characterized the xylanase genes in this assembled *R. cerealis* genome, investigated their expression patterns, and characterized the functional role of one xylanase, designated as RcXYN1, in fungal pathogenicity. The results provide evidence for RcXYN1 as an effector of *R. cerealis* during the fungal pathogenesis process in wheat.

## 2. Results

### 2.1. Global Identification of Xylanase Proteins from theR. cerealisGenome Sequence

**Figure 1:**
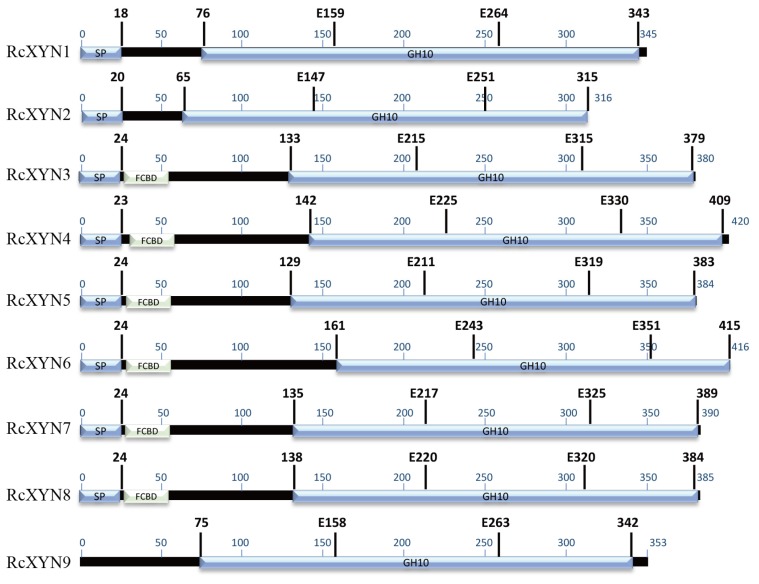
Predicted functional domains present in the GH10 family xylanases of *R. cerealis*. The region conserved in all the proteins is shown in blue. The glutamate responsible for catalysis is indicated by the letter E followed by a number that shows its position in the sequence. FCBD: fungal-type cellulose-binding domain.

Nine GH10 domain-containing xylanase proteins were identified in the proteome predicted from the *R. cerealis* genome sequence (unpublished). None of GH11 family proteins were detected. These nine GH10proteins, designated as RcXYN1–RcXYN9, all contained the GH10 domain. In addition, RcXYN3–RcXYN8 contained a carbohydrate-binding module 1 (CBM1) domain (fungal-type cellulose-binding domain (FCBD) in Figure 1) (Table 1, Figure 1). Of these nine xylanases, RcXYN1–RcXYN4 were predicted to be secreted proteins based on the presence of a typical signal peptide and the absence of a transmembrane domain (TMHs) (Table 1). Using DNAMAN and SMART software, we found that the shortest xylanase protein, RcXYN2, consisted of 316 amino acid (aa) residues and the longest, RcXYN4, had 420 aa residues. Across RcXYN1–RcXYN9, the lengths of the GH10 domains ranged from 294 to 315 aa residues. The predicted molecular weights/isoelectric points (pI) of all the xylanase proteins ranged from 33.43 kD/6.43 to 45.09 kD/9.32, respectively (Table 1).

### 2.2. Multiple Sequence Alignment and Intron–Exon Organization

**Figure 2:**
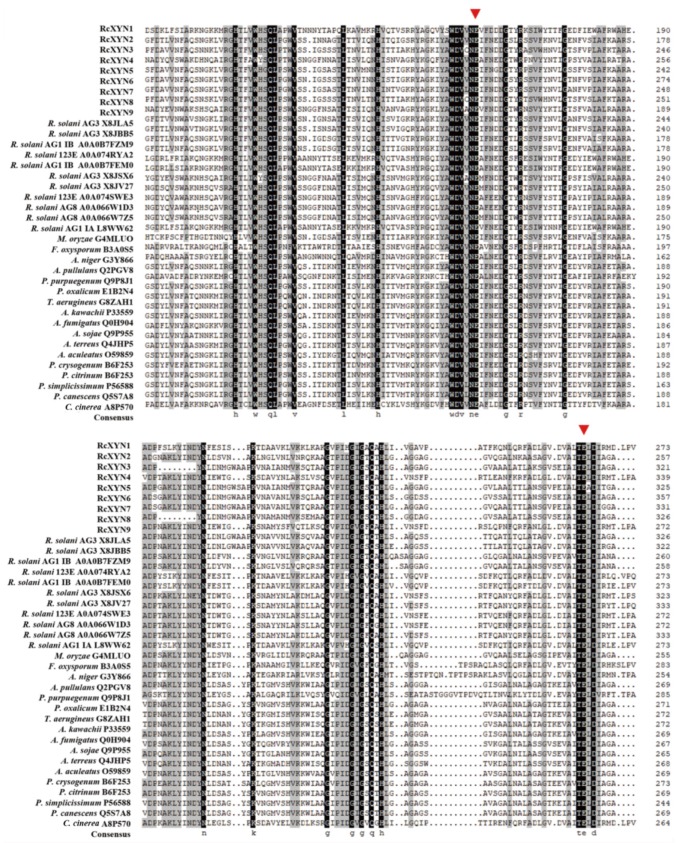
Alignment of amino acid sequences of the active site regions of the 37 GH10 family xylanases. Among them, there were nine GH10 xylanases from *R. cerealis*, 11 GH10 xylanases from *Rhizoctonia solani* (123E, AG1 IA, AG1 IB, AG2-2IIIB AG3, AG8), and 17 GH10 xylanases from 17 other fungi (*Magnaporthe oryzae, Fusarium oxysporum, Aspergillus niger, Aureobasidium pullulans, Penicillium purpurogenum, Penicillium oxalicum, Talaromyces aerugineus, Aspergillus kawachii, Aspergillus fumigatus, Aspergillus sojae, Aspergillus terreus, Aspergillus aculeatus, Penicillium crysogenum, Penicillium citrinum, Penicillium simplicissimum, Penicillium canescens,* and *Coprinopsis cinerea*). Red triangles represent the activity sites of these enzymes.

We conducted a protein sequence alignment among all nine GH10 xylanases from *R. cerealis* and 28 comparative GH10 xylanases from 18other fungi. Two conserved E (glutamate) residues for the xylanase catalysis were found in all these aligned sequences (Figure 2). With the exception of RcXYN9, all these enzymes possessed a signal peptide with a length of 18–24 aa residues (Figure 1). The conserved regions of these proteins in GH10 family ranged from 294 to 315 aa residues. Mature proteins of these GH10 xylanases had 301–389 aa residues. Because of heterogeneity in the number of amino acids, for these GH10 xylanases, the positions of the first glutamate were found between 158 and 225 aa residues and the second between 251 and351 aa residues. Multiple sequence alignment results showed that the identity among the amino acid sequences encoded by the nine *RcXYN* genes was 59.62% (Figure S1A).

All the genes of predicted GH10 xylanases in *R. cerealis* had different intron–exon patterns in relation to both the position and number of introns. Based on the number of introns, the xylanases were segregated into four patterns, pattern1 (RcXYN1), pattern 2 (RcXYN3, RcXYN8, and RcXYN9), pattern 3 (RcXYN5, RcXYN6, and RcXYN7), and pattern 4 (RcXYN2 and RcXYN4), containing 4, 7, 8, and 10 introns, respectively. Furthermore, significant differences in size (41-1326 bp) between the exons were observed. These results showed that the different xylanases had variable and complex intron–exon structures (Figure S1B).

### 2.3. Phylogenetic Analysis

**Figure 3:**
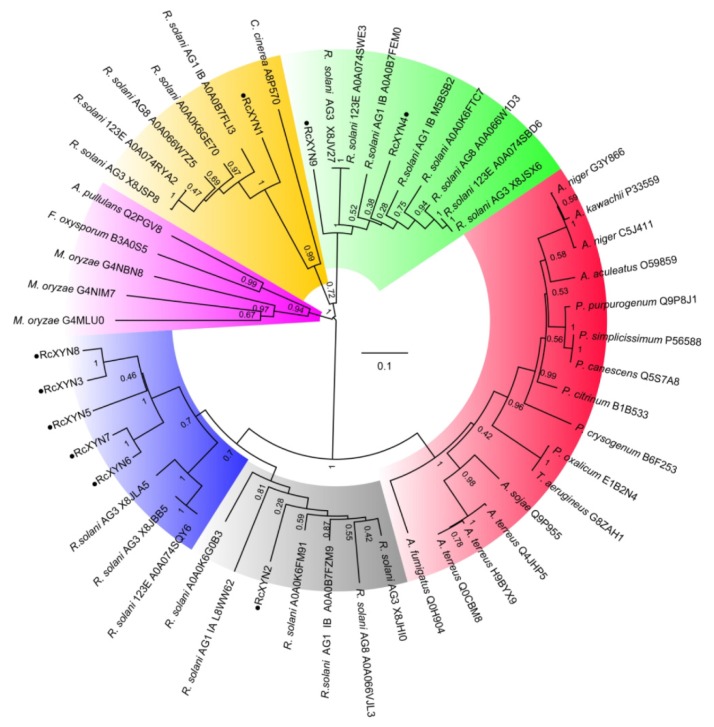
Phylogenetic relationships between GH10 xylanases from *R. cerealis* and other fungi. The phylogeny was constructed by Mega 7.0 using neighbor-joining method (parameters: 1000 bootstraps). Yellow represents group 1; Green represents group 2; Red represents group 3; Grey represents group 4A; Blue represents group 4B; Pink represents group 5.

To examine the relationship between the GH10 xylanases of *R. cerealis* and those from other fungal organisms, we constructed a phylogenetic tree based on the multiple sequence alignment of the 9 RcXYN protein sequences and 44 xylanases (GH10) from 18 other fungi (Figure 3). The phylogenetic tree divided these xylanases into five groups. Group 4 was further divided into the two subgroups 4A and 4B. In our analysis, RcXYN2 was placed in Group 4A (grey), while RcXYN3, RcXYN5-RcXYN8, and three xylanases from *R. solani* constituted Group 4B (blue). RcXYN1 was in Group 1(yellow), but RcXYN4 and RcXYN9 were clustered in Group 2 (green). None of the RcXYN proteins from *R. cerealis* were clustered in Group 3 (red) and 5 (pink). Multiple sequence alignment results show that the identity among the amino acid sequences encoded by the *RcXYN3* and *RcXYN5-8* genes was 80.10%. Coupled with the result inferred from the phylogenetic tree, RcXYN3 and RcXYN5–8 should be paralogs.

### 2.4. Expression Analysis of Xylanase Genes

**Figure 4:**
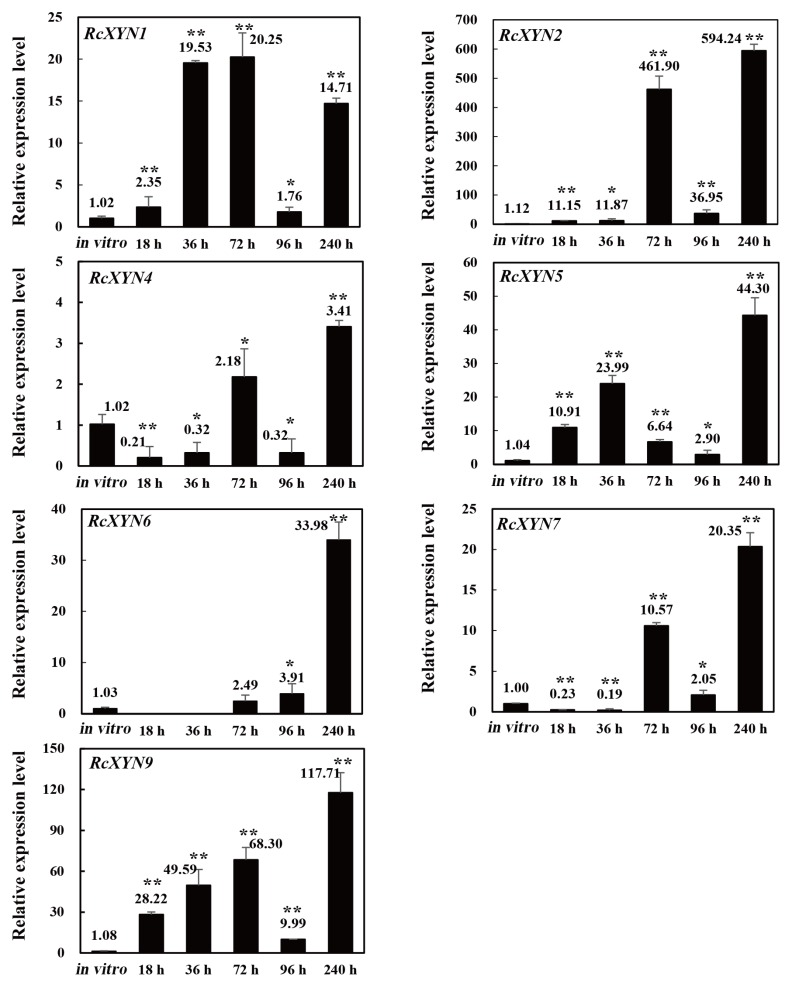
Expression patterns of the nine *RcXYN* genes in *R. cerealis* during the infection process of wheat stems. The *R.cerealis Actin* gene was used as an internal control to normalize cDNA. The expression of each xylanase gene was investigated during the infection process of wheat stems, including five different infection time points (18, 36, 72, 96, and 240 h after inoculation, hai) and compared to that of mycelia cultured in vitro. Error bars were calculated based on three replicates. * and ** indicate significant differences between *R. cerealis*-inoculated samples and in vitro samples (*t*-test; * *p* < 0.05 and ** *p* < 0.01).

Real-time quantitative PCR(RT-qPCR) expression analysis of *RcXYN1*–*RcXYN9* genes was investigated during the infection process over five time points (18, 36, 72, 96, and 240 h after inoculation, hai) and compared to expression in mycelia during in vitro culturing. The nine genes exhibited distinct expression patterns during the compatible interaction between *R. cerealis* and wheat (Figure 4). Transcriptional levels of *RcXYN1*, *RcXYN2*, *RcXYN5,* and *RcXYN9* were significantly up-regulated at five different times post *R. cerealis* inoculation when compared to mycelia in vitro. The transcriptional levels of *RcXYN6* and *RcXYN7* were up-regulated at 72, 96, and 240 hai, and showed a marked up-regulation at 240 hai compared with other time points. The transcriptional level of *RcXYN4* showed little change (<four-fold) at every time point. The *RcXYN3* and *RcXYN8* were not induced during the fungal infection of wheat, implying that these two xylanase genes might not participate in the infection process.

### 2.5. RcXYN1 Induces Necrosis andCell Death in Wheat and N. benthamianaLeaves

Heightened expression during the early infection stages suggests important role of *RcXYN1*, *RcXYN2*, *RcXYN5*, and *RcXYN9* in the pathogenicity of *R. cerealis*. The two putative secreted proteins RcXYN1 and RcXYN2 were selected to investigate their potential roles in pathogenicity. Sequence identity between RcXYN1 and RcXYN2 was 39.71%. The RcXYN1 protein shared 76.52% sequence identity with an unknown functional GH10 xylanase in *R. solani* AG1 IB with GenBank Accession Number CEL57784.1. The RcXYN2 protein shared 81.07% sequence identity with another unknown-functional GH10 xylanase in *R. solani* AG2-2IIIB with GenBank Accession Number CUA67331.1.

**Figure 5:**
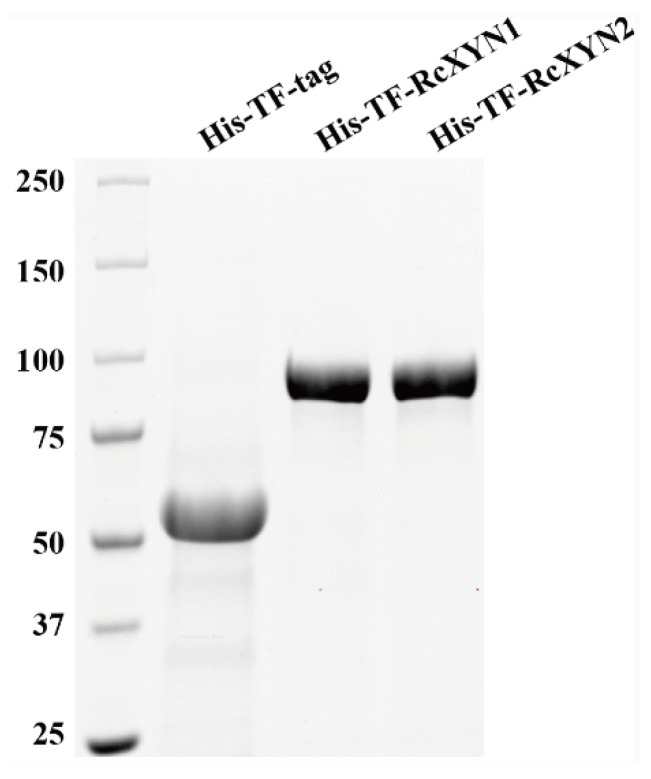
Heterologous expression of RcXYN1 and RcXYN2 in *Escherichia coli*. Sodium dodecyl sulphate-polyacrylamide gel electrophoresis (SDS-PAGE) patterns of the purified His-TF-RcXYN1 and His-TF-RcXYN2. His-TF-tag is the control sample.

To obtain purified RcXYN1 and RcXYN2 proteins, the *RcXYN1* and *RcXYN2* genes were subcloned and fused into the 3′ terminal of Trigger Factor (TF) in the pCOLD-TF expression vector, respectively. Then, the resulting recombinant His-TF-RcXYN1 protein and His-TF-RcXYN2 were expressed in *Escherichia coli*. After purification, the recombinant His-TF-RcXYN1 and His-TF-RcXYN2 proteins were examined through sodium dodecyl sulfate-polyacrylamide gel electrophoresis (SDS-PAGE). The purified recombinant proteins His-TF-RcXYN1 and His-TF-RcXYN2 migrated as single bands with estimated molecular masses of 86 kDa and 81 kDa on SDS-PAGE, respectively (Figure 5).

**Figure 6:**
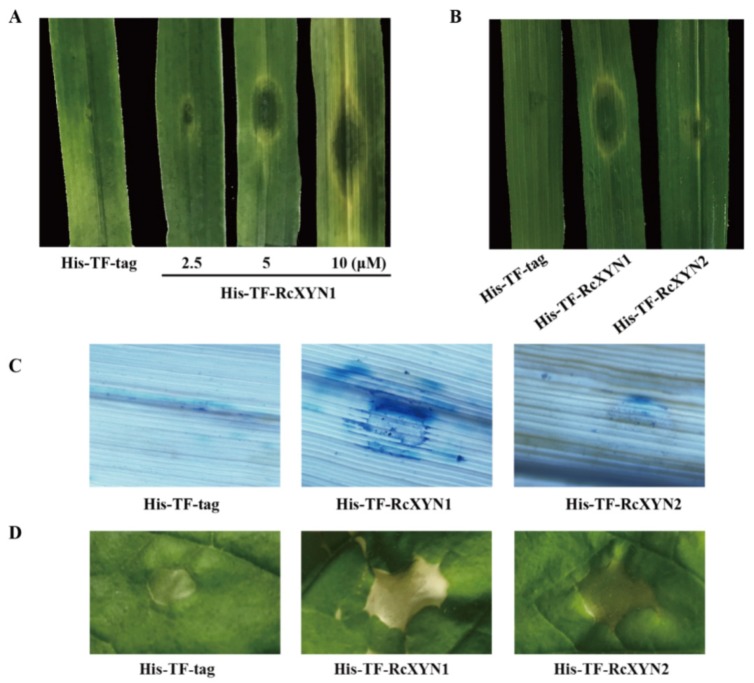
The necrosis and cell death induced by RcXYN1 in wheat and *Nicotiana benthamiana* leaves. (**A**) Wheat leaves 3 days post infiltration with the His-TF-RcXYN1 (2.5, 5, and 10 μM) and the His-TF-tag solution (5 μM) as control. (**B**) Wheat leaves 3 days post infiltration with the His-TF-RcXYN1 (5 μM) and His-TFRcXYN2 (5 μM). (**C**) Trypan blue staining of wheat leaves treated with His-TF-RcXYN1 (5 μM). Dead wheat leaf cells were stained by trypan blue. (**D**) *N. benthamiana* leaves infiltrated with His-TF-RcXYN1 (5 μM) and His-TF-RcXYN2 (5 μM).

The purified His-TF-RcXYN1 or His-TF-tag proteins were individually infiltrated into wheat leaves at concentrations of 2.5, 5, or 10 μM to test the ability to induce cell death. As shown in Figure 6A, necrosis and/or chlorosis were clearly observed in the His-TF-RcXYN1 infiltrated areas of the wheat leaves at three days after infiltration with all three concentrations, whereas infiltrations with5 μMHis-TF-RcXYN2 and His-TF-tag did not cause any visible necrotic symptoms (Figure 6B). In addition, trypan blue staining showed the occurrence of plant cell death in the necrotic areas induced by His-TF-RcXYN1 on wheat leaves (Figure 6C). We further tested the induced cell death-inducing activity of His-TF-RcXYN1 and His-TF-RcXYN2 in *N. benthamiana*. His-TF-RcXYN1 induced necrosis/chlorosis and cell death of the infiltrated leaves, but the His-TF-RcXYN2 and His-TF-tag control failed (Figure 6D).

### 2.6. RcXYN1 Can Induce H_2_O_2_ Accumulation in Infiltrated Plant Leaves

**Figure 7:**
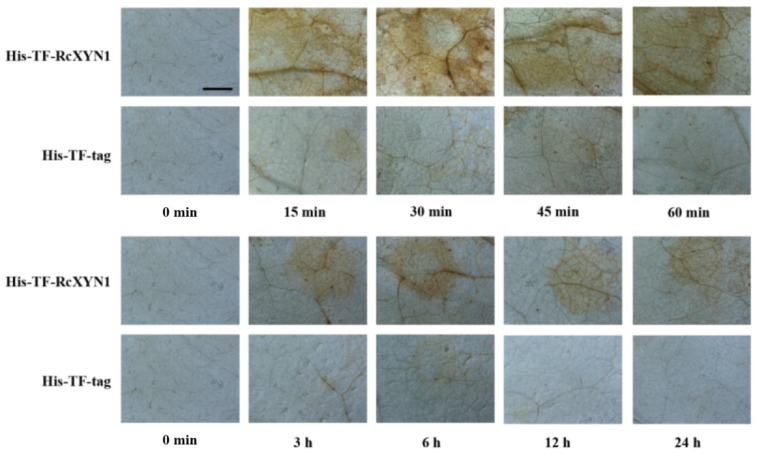
Microscopic observation of H_2_O_2_ accumulation in RcXYN1-infiltrated *N. benthamiana* leaves. H_2_O_2_ accumulation (as indicated by diaminobenzidine staining) appeared in the veins and stomata of His-TF-RcXYN1-infiltrated leaves but not in leaves treated with 5 μM His-TF-tag solution. Bar, 2 mm.

H_2_O_2_, a major type of ROS, often is correlated with cell death [35,36]. We examined if treatment of RcXYN1 could induce H_2_O_2_ production in the plant leaves. *N. benthamiana* leaves were infiltrated with His-TF-RcXYN1 and the control His-TF-tag, respectively, and then collected at different time points for diaminobenzidine (DAB) staining. As shown in Figure 7, H_2_O_2_ accumulation was detected at certain time points in *N. benthamiana* leaves infiltrated with His-TF-RcXYN1, mainly concentrated in the veins and stomata. No obvious DAB staining was observed for the infiltration with the His-TF control leaves. Greater H_2_O_2_ accumulation occurred at 15 min to 45 min after His-TF-RcXYN1 infiltration and with a significant decrease at 3 h and later time points (Figure 7), suggesting that ROS generation induced by RcXYN1 may be an early event.

### 2.7. RcXYN1 Contributes to Pathogenicity During R. cerealisInfection toWheat

**Figure 8:**
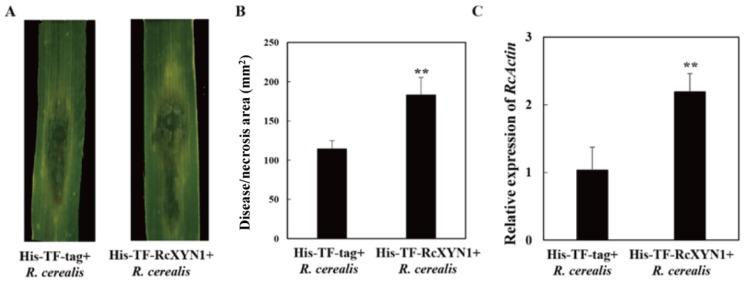
Contribution of RcXYN1 to pathogenicity in *R. cerealis* infection of wheat. (**A**) His-TF-RcXYN1 enhanced pathogenic phenotypes of *R. cerealis* in wheat leaves. Pictures of the lesions were taken at 3 days post inoculation with the fungus and the lesion area was measured; (**B**) Disease severity measured as area of necrosis induced by *R. cerealis* mycelia on leaves shown in (**A**); (**C**) RT-qPCR measurement of the pathogen and wheat RNA ratios were used to determine the *R. cerealis* relative biomass in infected plant tissues shown in (**A**). Three independent biological replicates were conducted. Error bars were calculated based on three replicates. ** indicates a significant difference between His-TF-RcXYN1 treatment and His-TF-tag treatment (*t*-test; *p* < 0.01).

To determine the pathogenicity role of RcXYN1, liquid culture-derived mycelia of *R. cerealis* Rc207 were inoculated on the surface of wheat leaves pre-infiltrated with His-TF-RcXYN1 or His-TF. Subsequently, disease development was recorded over time by measuring water-soaked areas. At 2 dpi, water-soaked lesions were clearly observed in *R. cerealis* inoculated leaves pretreated with the His-TF-RcXYN1 or His-TF. However, disease development was faster and the disease lesions were larger in leaves pretreated with His-TF-RcXYN1 than those pretreated with His-TF control (Figure 8A). Statistical analyses also revealed a significant difference between His-TF-RcXYN1 and His-TF control (Figure 8A,B). Furthermore, RT-qPCR examination on the transcription of *R. cerealis Actin* in the infected plant tissues exhibited that the fungal biomass in the His-TF-RcXYN1-pretreated leaves was significantly greater than that in the His-TF-pretreated control leaves (Figure 8C).

## 3. Discussion

In this study, we identified a total of nineGH10 family xylanases from the sequenced *R*. *cerealis* genome, characterized their gene structures and expression patterns, and investigated their possible roles in fungal pathogenicity. *RcXYN1*, one of the xylanase genes, was found to be expressed at high levels during the whole infection process and was able to induce necrosis/cell death in wheat and *N. benthamiana*, and stimulate ROS accumulation in *N. benthamiana.* To our knowledge, this is the first investigation on fungal pathogenesis in *R. cerealis* focusing on GH10 xylanase genes.

A total of nine non-redundant GH10 xylanase proteins were identified from *R. cerealis* genomic sequences, where five GH10 xylanase genes exist in *R. solani*AG8 and two GH10 xylanase genes are present in *R. solani*AG1 IA [15,34]. These GH10 enzymes of *R. solani*AG8 and *R. solani*AG1 IA have not been analyzed in detailed yet. However, no member belonging to GH11 family was found in the genomes of *R. cerealis* or *R. solani*AG1 IA and AG8. Such a fact may imply reliance in *Rhizoctonia* on GH10 family members for xylan degradation during the infection process of cereal host plants. Here, all the RcXYN1–RcXYN9 proteins include two conserved glutamate residues within the active motif in the GH10 domain. Previous papers documented that in other pathogenic fungi, xylanase proteins (GH10 and GH11) used the same double catalytic mechanism, which involve two highly conserved glutamate residues within the active site, one acting as an acid/base catalyst and the another as a nucleophile [37,38].

Although *R. cerealis* has nine xylanase genes, their expression patterns were quite different during the infection process over five time points (18, 36, 72, 96, and 240 h after inoculation, hai) compared to expression in culturing mycelia in vitro. Transcriptional levels of *RcXYN6* and *RcXYN7* were up-regulated at 72, 96, and 240 hai, and sharply enhanced at late infection time (240 hai), while *RcXYN3* and *RcXYN8* had no detectable expression at all five infection time points in comparison to the expression in culturing mycelia in vitro. The transcriptional level of *RcXYN4* showed little differential change (<four-fold) at every time point. The results implied that *RcXYN3, RcXYN4,* and *RcXYN8* might not participate in the infection process. Interestingly, *RcXYN1*, *RcXYN2*, *RcXYN5*, and *RcXYN9* expressed at high levels even from the beginning infection to wheat and maintained the high transcriptional levels for all the tested time points (the whole infection process to wheat). Early expression has also been observed for some xylanase genes from other fungal pathogens [25,39]. Induced expression at the early infection time suggested the importance of *ppxyn1* and *ppxyn2* in the pathogenicity of *P. parasitica* [25]. Similarly, transcriptions of both GH10 members from *B. cinerea* were the most abundant at the early stage, and continued increasing throughout the infection process [39].

Successful infection relies on the secretion of effector proteins with functions that facilitate host colonization, such as altering the structure and function of host cells [40,41,42]. Necrotrophic fungal pathogens utilize an array of effectors to induce plant cell death which may facilitate the growth of the necrotrophic pathogens [43]. Several xylanases from other fungal pathogens were also shown to have activity as necrotrophic effectors. For instance, ppxyn1 and ppxyn2 have been shown to play a crucial role in the infection process of *P. parasitica* [25]. In *B. cinerea,* BcXyl1, acting as an effector, is able to induce plant defense responses and contributes to pathogenicity [44]. In the current research, RcXYN1–RcXYN4, four xylanases in *R. cerealis,* were predicted to be secreted proteins, implying their potential as a necrotrophic effector. Considering these previous reports and the expressional patterns, we speculate that the predicted secretion proteinsRcXYN1 and RcXYN2may be involved in all infection and colonization stages of *R. cerealis*. Indeed, our functional dissection results proved that RcXYN1 is capable of inducing cell death at the treated plants. More importantly, application of the purified RcXYN1 together with *R. cerealis* led to significantly higher level of the disease in wheat leaves than application of the fungus alone. Therefore, it is likely that RcXYN1 functions as a fungal effector to induce plant cell death facilitating the late colonization of the necrotrophic fungal pathogen. The RcXYN2 protein could not cause obvious necrosis/cell death, suggesting that RcXYN2 was not a pathogenicity factor, and maybe play other roles during the infection of *R. cerealis* to wheat. To determine whether RcXYN2 still has its xylanase function, enzymatic activity for RcXYN1 and RcXYN2 recombinant proteins will be assayed in future.

In conclusion, we identified nine xylanase genes from the *R. cerealis* genome. They exhibited different expression patterns during the fungal infection process of wheat. RcXYN1, a putative secreted xylanase in *R. cerealis*, was demonstrated to trigger plant cell death and H_2_O_2_ accumulation, and to contribute to the fungal pathogenicity on wheat. Thus, *RcXYN1* likely is an important gene resource for improving resistance of wheat and other crop plants to *R. cerealis* through host-induced gene silencing strategy. These current findings facilitate a better understanding of the pathogenesis mechanisms of the *R. cerealis* in the fungus–wheat interactions.

## 4. Materials and Methods

### 4.1. Fungal Strain, Plant Materials, and Growth Conditions

The necrotrophic fungus *R. cerealis* isolate Rc207, a highly aggressive strain collected in Northern China, was kindly provided by Professor Jinfeng Yu at Shandong Agricultural University [5]. This strain was maintained on potato dextrose agar (PDA) at 4 °C. To conduct pathogenicity tests, subcultures were made on new PDA plates or potato dextrose liquid culture, which were then cultivated at 25 °C for 10 days before inoculation.

Wheat cultivar Wenmai6 was highly susceptible to *R. cerealis* infection. Wheat plants were grown in a greenhouse under a 13-h light (~22 °C)/11-h dark (~10 °C) regime. At their tillering stage, the second base sheath of each wheat plant was inoculated with *R. cerealis* isolate Rc207 using the toothpick inoculated method [45]. The stems of wheat at five different infection time points (18, 36, 72, 96, and 240 hai) with *R. cerealis* Rc207 were sampled.

*Nicotiana benthamiana* plants were grown under standard glasshouse conditions at 25 °C with a 12-h light and 12-h dark regime.

### 4.2. Identification of GH10 Xylanase Genes in Rhizoctonia Cerealis

The software Hmmer was used to annotate the function of carbohydrate enzymes based on the carbohydrate related enzyme database. Members of the candidate xylanase gene family were identified using BlastP with an *E*-value less than 1e^−10^ from the *R. cerealis* Rc207 genome sequence. The codes indicating the enzyme classes were those defined by the CAZyme database [46]. Signal peptide and transmembrane domains were predicted with SignalP v4.0 [47] and TMHMM [48]. Putative proteins containing signal peptide and no transmembrane domains outside the signal peptide region were identified as secreted proteins. Multiple alignments were made with the DNAman software. Amino acid sequences employed for conserved residue analysis were as indicated. A neighbor-joining (NJ) tree was constructed using the program MEGA (version 7.0) based on the multiple sequence alignment of the 9 RcXYN protein sequences and 44 xylanases (GH10) from other fungi downloaded from the NCBI database (File S1).

### 4.3. DNA and RNA Extraction and cDNA Synthesis

Total genomic DNA was extracted from the mycelia of *R. cerealis* Rc207 using the modified cetyl-trimethyl-ammonium bromide extraction method [49]. Total RNAs from the *R. cerealis* Rc207 and the pathogen-inoculated stems or RcXYN1-treated leaves of wheat plants were extracted using the TRIzol reagent (Invitrogen, Life Technologies, Carlsbad, CA, USA), purified with RNase-free DNase I (Takara, Takara, Japan) [50]. Reverse transcription was performed by using a PrimeScript^TM^ RT Reagent Kit with gDNA Eraser (Takara, Takara, Japan) according to the manufacturer’s instruction.

### 4.4. Real-Time Quantitative PCR (RT-qPCR) Analysis

To examine transcripts of the xylanase genes during the infection process in wheat, fungal RNAs were extracted from the pathogen inoculated stems of wheat at 18, 36, 72, 96, and 240 h after inoculation (hai) with *R. cerealis* Rc207 and mycelia from *R. cerealis* Rc207 in vitro.

An ABI 7500 RT-PCR system (Applied Biosystems, Waltham, MA, USA) was used to conduct RT-qPCR following the procedure described in Dong et al. [51]. The relative expression of the target genes in *R. cerealis* were calculated using the 2^−ΔΔCT^ method [52]. The wheat *Actin* gene (*TaActin*) or the *R. cerealis Actin* gene (*RcActin*) were used as the internal reference. Three independent biological replications were evaluated. The specific RT-qPCR primers for each xylanase gene were designed based on the specific sequences using homologous sequence alignment to distinguish each other. All the primers in the study are listed in Table S1.

### 4.5. Heterologous Expression of RcXYN1 and RcXYN2

The full coding sequences of the *RcXYN1* and *RcXYN2* genes were sub-cloned into the *Bam*HI site of the pCOLD-TF vector and fused with the His-Trigger Factor protein (His-TF) tag, resulting in the expression vectors pCOLD-TF-RcXYN1 and pCOLD-TF-RcXYN2, respectively. The positive clones with the *His-TF-RcXYN1* and *His-TF-RcXYN2* recombinant genes were identified by means of gene-specific PCR and confirmed by sequencing of each respective gene. The resulting pCOLD-TF-RcXYN1 and pCOLD-TF-RcXYN2 fusion constructs were transformed into competent cells of *Escherichia coli* (*E. coli*) *BL21* (DE3), respectively. The recombinant proteins were expressed after treatment with 0.5 mM isopropyl-β-D-thiogalactopyranose at 16 °C for 19 h, and purified using Ni-NTA resin. Protein purity and molecular weight were determined by using SDS-PAGE (Bio-Rad, Hercules, CA, USA).

### 4.6. Cell Death-Inducing Activity of the RcXYN1 Protein

Cell death-inducing activity of the heterologous expressed proteins was assayed by infiltrating samples (25 µL) into detached leaves of two-month-old wheat plants [8,34,53]. To determine the minimum concentration required, 25 μL of a serially diluted protein solution (2.5, 5, or 10 μM) was infiltrated into wheat leaves. His-TF-tag protein was used as a negative control.

### 4.7. Diaminobenzidine (DAB) Staining for Detection of H_2_O_2_

Detection of hydrogen peroxide (H_2_O_2_) in plants was conducted following a previously described method based on DAB staining [54]. *Nicotiana benthamiana* leaves treated by His-TF-RcXYN1 were sampled, and then immediately vacuum-infiltrated with a solution of 1 mg mL^−1^ DAB-HCl (pH 3.8) at 25 °C. These were placed under darkness for 8 h and subsequently boiled for 5 min in 95% ethanol [55].

### 4.8. Application of the Purified RcXYN1 in Disease Assays

Following the test method of Lu et al., in a detached-leaf inoculation assay, fully expanded secondary leaves (at the tillering stage in two-month-old wheat Wenmai6) were infiltrated with His-TF-RcXYN1 (25 µL) [8]. Following protein infiltration for six hours, leaves were inoculated in the same location with 50 µL mycelial suspension of Rc207. The leaves were then placed in Petri dishes containing filter paper saturated with sterile distilled water and maintained under a 16-h day/8-h night regime at 25 °C. Leaf lesions were photographed at 3 days post inoculation (dpi) with Rc207, with lesion areas measured by length×width. Each experiment was performed with six leaves and repeated three times. The pathogen biomass was quantified by RT-qPCR and the primers specific to the *actin* gene of *R. cerealis* (*RcActin*) (Table S1). Three independent biological replicates were conducted.

## Figures and Tables

**Table 1:** Characteristics of nine glycoside hydrolase 10 (GH10) xylanase genes (*RcXYNs*) in *Rhizoctonia cerealis.*

Gene Name	Coding Sequence Size (bp)	aa	Molecular Weight (kD)	Signal P	pI	TMHs	Secreted Protein	CAZYmes
*RcXYN1*	1038	345	38.36	Yes	9.32	0	Yes	GH10
*RcXYN2*	951	316	33.42	Yes	9.22	0	Yes	GH10
*RcXYN3*	1143	380	39.54	Yes	7.68	0	Yes	GH10, CBM1
*RcXYN4*	1263	420	45.09	Yes	6.90	0	Yes	GH10, CBM1
*RcXYN5*	1155	384	39.82	Yes	8.57	1	No	GH10, CBM1
*RcXYN6*	1251	416	43.22	Yes	6.82	1	No	GH10, CBM1
*RcXYN7*	1173	390	40.13	Yes	7.67	1	No	GH10, CBM1
*RcXYN8*	1158	385	39.69	Yes	6.43	1	No	GH10, CBM1
*RcXYN9*	1062	353	38.48	No	7.89	0	No	GH10

## Supplementary Materials

The following are available online at https://www.mdpi.com/1422-0067/21/5/1812/s1, Figure S1: Sequence alignment among *RcXYN* proteins in *Rhizoctonia cerealis* andstructures of their coding genes. (A) Sequence alignment among nine *Rhizoctonia cerealis* xylanases proteins. (B) Exons and introns are indicated by black boxes and lines, respectively. The 5′-3′ scale indicates the DNA sequence size. The names of the *RcXYN* genes and intron-exon structures are indicated at the left and right sides, respectively. Table S1: All primers used in this study. File S1: Amino acid sequences of the 53 GH10 family xylanases from Rhizoctonia cerealis and other fungi (*Rhizoctonia solani, Magnaporthe oryzae, Fusarium oxysporum, Aspergillus niger, Aureobasidium pullulans, Penicillium purpurogenum, Penicillium oxalicum, Talaromyces aerugineus, Aspergillus kawachii, Aspergillus fumigatus, Aspergillus sojae, Aspergillus terreus, Aspergillus aculeatus, Penicillium crysogenum, Penicillium citrinum, Penicillium simplicissimum, Penicillium canescens, Coprinopsis cinerea*).
