# Supplementary material for: Global Characterization of GH10 Family Xylanase Genes in Rhizoctonia cerealis and Functional Analysis of Xylanase RcXYN1 During Fungus Infection in Wheat
Source: Int J Mol Sci. 2020 Mar 6;21(5):1812. doi: 10.3390/ijms21051812 (PMC7084588; doi:10.3390/ijms21051812)
Supplement: Supplementary file 1 [file ijms-21-01812-s001.pdf]

Supporting information

Table S1 All primers used in this study.

Primer Name	Sequence (5'-3')
RcXYN1-F	5′-CCACCTTCACTATGCTCG-3′
RcXYN1-R	5′-TACGTGGTACACAATTCCT-3′
RcXYN2-F	5′-ACTCGATTCTGCTACCAT-3′
RcXYN2-R	5′-AGGGAGCAACACTCTGTA-3′
RcXYN3-F	5′-GGGGATGCCAGTTAGACG-3′
RcXYN3-R	5′-TGTGCTGCGACGGTATT-3′
RcXYN4-F	5′-GCTCTTCTAATGGTCTCGCC-3′
RcXYN4-R	5′-GCTGGTATCCCAAACCGTAAT-3′
RcXYN5-F	5′-ACATTGGCTGACTTGGAC-3′
RcXYN5-R	5′-TCCGAAAGCACAATCTACAAA -3′
RcXYN6-F	5′-ATAGAATGGTAGGGACAAGAC-3′
RcXYN6-R	5′-AACAGGCAAAGCATTACAG -3′
RcXYN7-F	5′-AACTCGCATCTAACTAGGACA-3′
RcXYN7-R	5′-ATTTACTAGAAATTGAAAGAA -3′
RcXYN8-F	5′-CAACGGAGGACTAAATGC-3′
RcXYN8-R	5′-AAAGCATCATAGAGCCAATTC -3′
RcXYN9-F	5′-GCCCAATGGTTTGTTTC-3′
RcXYN9-R	5′-AGTAAATTCTTCATCCGG-3′
pCOLD-TF-RcXYN1-F	5′-ggtaccctcgagggatccATGCTCGCGCTCTCTGCTC-3′
pCOLD-TF-RcXYN1-R	5′-aagcttgaattcggatccTCATGCCCCCAAAGCCTTTC-3′
pCOLD-TF-RcXYN2-F	5′-ggtaccctcgagggatccATGCGCTTTACTGTTGCCAC-3′
pCOLD-TF-RcXYN2-R	5′-aagcttgaattcggatccTCAATTCAAAGCCGAGATGA-3′
TaActin-F	5'-CACTGGAATGGTCAAGGCTG-3'
TaActin-R	5'-CTCCATGTCATCCCAGTTG-3'
RcActin-F	5'-GCATCCACGAGACCACTTAC-3'
RcActin-R	5'-GCGTCCCGCTGCTCAAGAT-3'
QRcXYN1-F	5'-CCACCGAGTCTTACAACATCCA-3'
QRcXYN1-R	5'-CCACGCATCTTCTTCCCATTT-3'
QRcXYN2-F	5′-GGAGGCTTTGATACCTTGGTC-3′
QRcXYN2-R	5′-TGGCATTTCCGTCCGCAGAGC-3′
QRcXYN3-F	5'-TCTCAGACCGCCGCTGGCACTC-3'
QRcXYN3-R	5'-CCTTGGGCTTGAAGTTCGAGTCATA-3'
QRcXYN4-F	5′-CCACGCTCATCTCAGTCCTTC-3′
QRcXYN4-R	5′-TGGCAGTTGGGTCTACAGCAC-3′
QRcXYN5-F	5′-GGTGGTATCGGCTACAGTGGT-3′
QRcXYN5-R	5′-GACCGTCAAGTCCGCTACCTC-3′
QRcXYN6-F	5′-GTCGTAGTTCTCATGCCACAAGC-3′
QRcXYN6-R	5′-CCGCCTCCAGGATTAGGGTTA-3′
QRcXYN7-F	5′-CGTCGCCATCCTCAAGTCTGA-3′
QRcXYN7-R	5′-CTGACTGTGCCAGATGAAGGTG-3′
QRcXYN8-F	5'-CCTCAGTATGGCACAATGTTCTT-3'
QRcXYN8-R	5'-TGCATCTCCCCCACTCAGGTGA-3'
QRcXYN9-F	5'-GCTGCTCTGGGCGTTCTTGCT-3'
QRcXYN9-R	5'-TCGGACGGGTTTCCAGTGAGG-3'

A

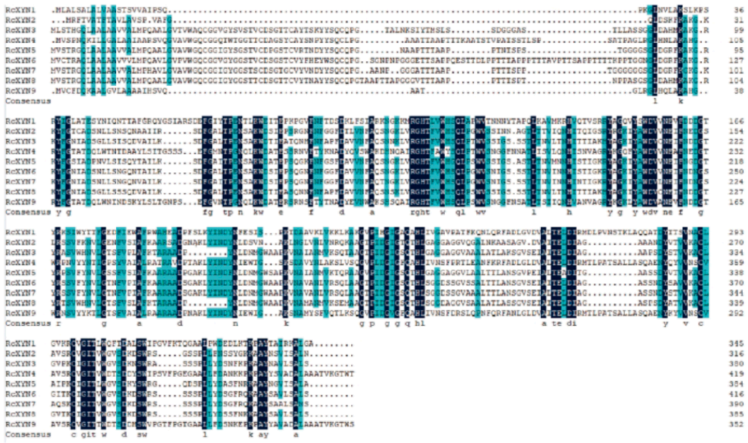

B

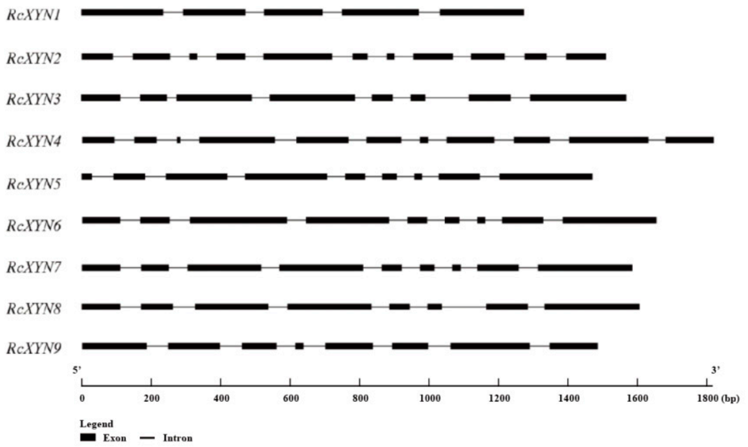

Figure S1 Sequence alignment among *RcXYN*proteins in *Rhizoctonia cerealis* andstructures of their coding genes. (A) Sequence alignment among nine *Rhizoctonia cerealis* xylanases proteins.(B) Exons and introns are indicated by black boxes and lines, respectively. The 5′-3′ scale indicates the DNA sequence size. The names of the *RcXYN* genes and intron-exon structures are indicated at the left and right sides, respectively.

File S1 Amino acid sequences of the 53 GH10 family xylanases from *Rhizoctonia cerealis* and other fungi (*Rhizoctonia solani*, *Magnaporthe oryzae*, *Fusarium oxysporum*, *Aspergillus niger*,*Aureobasidium pullulans*, *Penicillium purpurogenum*, *Penicillium oxalicum*, *Talaromyces aerugineus*, *Aspergillus kawachii*, *Aspergillus fumigatus*, *Aspergillus sojae*, *Aspergillus terreus*, *Aspergillus aculeatus*, *Penicillium crysogenum*, *Penicillium citrinum*, *Penicillium simplicissimum*, *Penicillium canescens*, *Coprinopsis cinerea*).
